# A Paper-Based Piezoelectric Accelerometer

**DOI:** 10.3390/mi9010019

**Published:** 2018-01-02

**Authors:** Yu-Hsuan Wang, Pengfei Song, Xiao Li, Changhai Ru, Giovanni Ferrari, Prabakaran Balasubramanian, Marco Amabili, Yu Sun, Xinyu Liu

**Affiliations:** 1 Department of Mechanical Engineering, McGill University, Montreal, QC H3A 0G4, Canada; vw0821@gmail.com (Y.-H.W.); pengfei.song@mail.mcgill.ca (P.S.); xil@stanford.edu (X.L.); giovanni.ferrari@mail.mcgill.ca (G.F.); prabakaran.balasubramanian@mail.mcgill.ca (P.B.); marco.amabili@mcgill.ca (M.A.); 2 Department of Mechanical and Industrial Engineering, University of Toronto, Toronto, ON M5S 3G8, Canada; sun@mie.utoronto.ca; 3 Research Center of Robotics and Micro System & Collaborative Innovation Center of Suzhou NanoScience and Technology, Soochow University, Suzhou 215021, China; rchhai@gmail.com

**Keywords:** paper-based sensors, microelectromechanical systems (MEMS), piezoelectric accelerometer, hydrothermal growth, zinc oxide nanowires

## Abstract

This paper presents the design and testing of a one-axis piezoelectric accelerometer made from cellulose paper and piezoelectric zinc oxide nanowires (ZnO NWs) hydrothermally grown on paper. The accelerometer adopts a cantilever-based configuration with two parallel cantilever beams attached with a paper proof mass. A piece of U-shaped, ZnO-NW-coated paper is attached on top of the parallel beams, serving as the strain sensing element for acceleration measurement. The electric charges produced from the ZnO-NW-coated paper are converted into a voltage output using a custom-made charge amplifier circuit. The device fabrication only involves cutting of paper and hydrothermal growth of ZnO NWs, and does not require the access to expensive and sophisticated equipment. The performance of the devices with different weight growth percentages of the ZnO NWs was characterized.

## 1. Introduction

Over the past three decades, there have been numerous advances made in microfabricated sensors and actuators. As the research and development on microelectromechanical systems (MEMS) progress, it is increasingly common to integrate materials other than silicon into MEMS transducers. Among these developments, piezoelectric materials in either bulk or thin film forms have been integrated into MEMS devices for a variety of applications such as acoustic resonators [1,2], quartz crystal microbalance [3,4], Surface acoustic wave (SAW)-based chemical and biochemical sensors [5,6], accelerometer [7,8,9,10] and ultrasonic transducers [11,12]. However, the microfabrication of these devices typically requires the access to sophisticated cleanroom equipment and tedious training, and the fabrication process is time-consuming and relatively expensive. It is desired to resort to MEMS designs that only involve simple and inexpensive fabrication techniques for device construction.

Besides solid-state materials, paper has also emerged as a substrate material for the development of flexible sensors and electronics as it offers some unique advantages such as low cost, excellent biodegradability and disposability, ease of fabrication, good printability and foldability, high flexibility and light weight. There have been significant advances in paper-based transducers and electronics [13,14], and various electronic devices have been developed such as radio-frequency identifications (RFIDs) [15,16], biosensors [17,18,19], MEMS [20,21,22], transistors [23,24], capacitors [25,26], flexible display [27,28], energy harvesting and storage [29,30], smart packaging [31] and so forth. In particular, efforts have been made on developing paper-based physical transducers such as nanogenerators [32,33] and strain sensors [34].

Applications such as smart packaging require vibration monitoring during transportation, and paper-based accelerometers could provide a suitable solution for seamless integration. There are only two designs reported in the literature that is capable of acceleration measurement using functionalized paper substrates [35,36]. Mahadeva et al. [35] designed a hybrid piezoelectric paper material with a large piezoelectric coefficient by anchoring barium titanate nanoparticles (NPs) to surface-functionalized wood fibres, and fabricated it into a paper accelerometer to demonstrate the potential of such piezoelectric hybrid paper material. The piezoelectric charges generated from the device during vibration were measured directly as the output signal, and no signal processing circuit was developed for converting the piezoelectric charge into an output voltage. Mazzeo et al. [36] filed a patent disclosing the design of capacitive accelerometers made from commercially-available metalized paper. Such a capacitive accelerometer consists a pair of parallel plates of metalized paper with one fixed and the other tethered by flexures. The acceleration-induced inertial force exerted on the tethered plate can be detected by measuring the change in capacitance of the parallel plates. Although this design is feasible in principle, the patent application does not include any experimental data of the accelerometer fabrication and testing and the device performance is not characterized.

**Figure 1:**
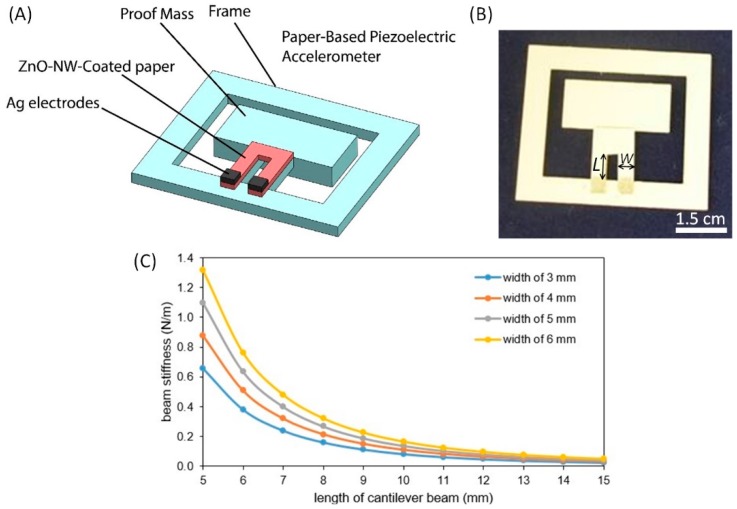
Design of the paper-based accelerometer. (**A**) Schematics of the paper-based accelerometer. (**B**) Photograph of a prototype device. (**C**) The stiffness of the two parallel beams as a function of the parallel beam width (*W*) and length (*L*).

In this work, we take a different approach to the development of a fully-functional, paper-based piezoelectric accelerometer based on zinc oxide nanowire (ZnO NW)-coated cellulose paper. Through a facile hydrothermal approach, we grow ZnO NWs on a piece of cellulose chromatography paper, which renders the paper piezoelectric. We use the ZnO-NW-coated paper as a strain sensing element and attach it on top of a set of two parallel cantilever beams (Figure 1A). When a proof mass connected at the ends of the two cantilever beams experiences an acceleration, the resultant inertial force bends the beams and causes mechanical strain on the ZnO-NW-coated paper. The piezoelectric charges produced from the ZnO-NW-coated paper are finally converted into a voltage output via a charge amplifier circuit. We perform experiments to calibrate the paper-based accelerometer and examine the relationship of the amount of ZnO NWs grown on the strain sensing paper and the sensor performance.

## 2. Experimental Methods

### 2.1. Design of the Paper-Based Piezoelectric Accelerometer

As illustrated in Figure 1A,B, the paper-based accelerometer consists of a paper frame that serves as the structural support of the sensor, a proof mass connected to one side of the frame through two parallel cantilever beams, a piece of U-shaped ZnO-NW-coated chromatography paper attached on top of the cantilever beams (for sensing acceleration-induced strains) and two silver electrodes screen printed at the two ends of the U-shaped ZnO-NW-coated paper (for collection of piezoelectric charges). The ZnO NWs are hydrothermally grown from a layer of ZnO NPs seeded on cellulose microfibers of the chromatography paper (see Section 2.2 for details). When the frame of the paper-based accelerometer is firmly attached to a vibration source, the proof mass undergoes vibration and the ZnO-NW-coated paper attached on the cantilever beams thus experiences mechanical deformation. Due to the piezoelectric property of the ZnO-NW-coated paper, electric charges are generated upon the applied mechanical deformation and collected through the silver electrodes at the roots of the U-shaped paper. Figure 1C shows the calculation results of the total stiffness of the parallel beams as a function of the width and length of each individual beam, which are obtained based on the stiffness equation of a cantilever beam. In the calculation, the beam thickness was fixed at 410 μm (total thickness of the parallel cantilever beam and the ZnO-NW-coated paper) and the Young’s modulus of the paper material was *E* = 1.59 GPa. For device prototyping, the length (*L*) and width (*W*) of the parallel beams were set to be 10 mm and 5 mm, respectively. Other beam dimensions could also be used if a different total beam stiffness is desired.

The piezoelectric output from the paper-based accelerometer could be attributed to two piezoelectric mechanisms. (i) As the cantilever beams experience acceleration-induced deformations, the root/seed layer of ZnO coated on the paper microfibers is deformed, which produces electric charges. (ii) As the cellulose microfibers of the ZnO-NW-coated paper are deformed, adjacent ZnO NWs grown on them contact and bend against each other, generating electric charges and transporting these charges through the root layer of ZnO and the network of contacted ZnO NWs. Further efforts are needed on multiscale mechanical modelling of the hierarchical structure of the ZnO-NW-coated paper, in order to better understand deformations of the cellulose microfibers and the individual ZnO NWs and thus decipher the piezoelectric strain sensing principle of the ZnO-NW-coated paper.

### 2.2. Hydrothermal Growth of ZnO NWs on Cellulose Papers

A hydrothermal approach was employed to grow ZnO NWs on Whatman^®^ Grade 1 chromatography paper (180 μm thick, GE Healthcare, Mississauga, ON, Canada), which has been previously reported in detail [37]. Briefly, the growth process includes two steps: (i) the seeding of ZnO NPs on paper and (ii) the growth of ZnO NWs from the seeded NP layer. In the first step, uniform coating of ZnO NPs on the paper substrate was achieved through repeated dipping of the paper substrate into a colloidal solution of ZnO NPs. This provides a starting point for the subsequent growth process. To prepare the ZnO-NP solution, zinc acetate dihydrate (ZAD; 20 mL, 4 mM) and sodium hydroxide (NaOH; 20 mL, 4 mM) solutions were separately prepared in ethanol (200 proof) at 70 °C, stirred at 600 rpm on a stirring hotplate (Isotemp™ stirring hotplate, Fisher Scientific). Another 20 mL of ethanol was then added to the ZAD solution with constant stirring for further dilution. Finally, the solutions of ZAD and NaOH were mixed and placed in an oven at 60 °C for 2 h to form a colloidal solution of ZnO NPs. The size of the ZnO NPs was characterized to be 3.3 ± 0.6 nm (*n* = 50). After the seeding solution was cooled down to room temperature, three 30 mm × 30 mm square pieces of Whatman^®^ Grade 1 chromatography paper were immersed into the solution for 3 min and dried at 86 °C for another 3 min. This dipping and drying process was repeated four times and each time the side of the paper piece facing up was alternated so the effect of gravity on the excessive ZnO-NP seeding solution left on either side of the paper substrate was canceled out. At the end of the first step, a uniformly coated ZnO-NP film was formed on the surfaces of the individual cellulose microfibers of the paper substrate.

In the second step of the hydrothermal growth process, the ZnO-NP-coated paper substrates were immersed in an aqueous solution of zinc nitrate hexahydrate (ZNH; 25 mM) and hexamethylenetetramine (HMTA; 12.5 mM) in a 50 mL flask with stopper. The ZNH is to provide the zinc ions (Zn^2+^) and HMTA is to supply the hydroxyl ions that react with Zn^2+^ ions to form ZnO [37]. The flask containing the paper substrates and the growth solution was placed inside an oven at 86 °C for 1–4 h. To optimize the efficiency of the hydrothermal growth, ammonium hydroxide (AH) was added to the growth solution at 0.465 M to suppress the homogeneous nucleation of ZnO NWs [38]. We adjusted the length of the ZnO NWs grown on paper by changing the growth time. The longer the growth is performed, the longer the ZnO NWs are obtained. We found that the diameter of the ZnO NWs did not change significantly with the growth time. We used the weight growth percentage (relative increase in the weight of a dried paper substrate after the growth process) to quantify the amount of ZnO NWs grown on a paper substrate using a precision balance (Practum513-1S, Sartorius, Göttingen, Germany; resolution: 0.001 g), which is approximately proportional to the length of the ZnO NWs.

### 2.3. Fabrication of the Paper-Based Piezoelectric Accelerometer

To fabricate a paper-based accelerometer, a laser cutter was used to cut out a device frame using card stock paper (thickness: 230 μm, Staples, Framingham, MA, USA), which consists of a rectangular frame connected to a rectangular proof mass at the frame center through a set of two parallel cantilever beams. Three layers of card stock paper were attached to the proof mass using double-sided adhesive tape, leading to a total mass of 61 ± 1.1 mg (*n* = 9). Then, a U-shaped ZnO-NW-coated paper was attached to the parallel cantilever beams using double-sided tape. Finally, silver ink (E1660, Ercon, Wareham, MA, USA) was manually screen-printed onto two ends of the ZnO-NW-coated paper to form two electrodes of 2.5 mm × 2.5 mm, and the ink was dried on a hotplate at 80 °C for an hour. Figure 1B shows a paper-based accelerometer prototype after fabrication. The silver electrode formed an Ohmic contact with the ZnO-NW-coated paper [37]. A charge amplifier circuit was developed to convert and amplify the electric charges generated from the ZnO NWs into a voltage signal as the sensor readout.

### 2.4. Design of the Charge Amplifier Circuit

The piezoelectric charge output from the ZnO-NW-coated paper, caused by acceleration-induced mechanical deformations, is small and can be measured by a precision potentiostat (PGSTAT302N, Metrohm, Herisau, Switzerland) as a current on the order of a few nanoamperes. To facilitate the signal measurement from the paper-based accelerometer, we designed a custom-made charge amplifier circuit that performs two tasks: (i) to convert piezoelectric charges generated from the ZnO NWs into a voltage signal; and (ii) to amplify the voltage signal to a level suitable for direct data acquisition (DAQ) using a standard DAQ board (NI USB-6341, National Instruments, Austin, TX, USA). All the experiments in this work were performed using the charge amplifier circuit connected to the DAQ board, unless otherwise indicated. In order to further enhance the portability of the experimental setup, another option is to replace the DAQ board with a portable microprocessor circuit for data collection.

**Figure 2:**
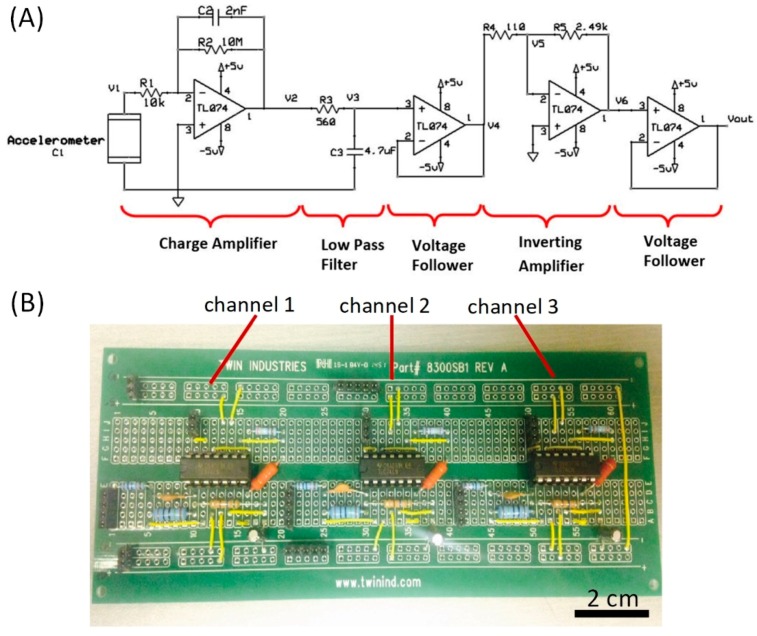
(**A**) Diagram and (**B**) photograph of the charge amplifier circuit.

Figure 2A shows the schematic layout of the charge amplifier circuit. It consists of a charge amplifier for converting electric charges into a voltage signal, a low-pass filter with a cut-off frequency of 60.4 Hz to remove high-frequency noises, a voltage follower that acts as a buffer to prevent loading effect, an inverting amplifier to amplify the voltage signal and finally another voltage follower to avoid loading effect before sensor readout by the DAQ board. Figure 2B is a picture demonstration of three identical charge amplifier circuits for multiple sensor readouts.

The overall transfer function of the charge amplifier circuit is:

(1) $$H\left(s\right)\;=\;\frac{V_{\mathrm{out}}}{V_{1}}\;=\;G_{\mathrm{DC}}\left(\frac{sR_{1}C_{1}}{1+\frac{s}{2\pi f_{L}}}\right)\left(\frac{1}{1+\frac{s}{2\pi f_{H}}}\right)\left(\frac{1}{1+\frac{s}{2\pi f_{c}}}\right),$$

where *G*_DC_ is the circuit direct-current (DC) gain: $G_{\mathrm{DC}}\;=\;\frac{R_{2}}{R_{1}}\times \frac{R_{5}}{R_{4}}\;=\;\frac{10\;MΩ}{10\;kΩ}\times \frac{2.49\;kΩ}{110\;Ω}\;=\;22636$, *f*_L_ and *f*_H_ are the lower and higher cut-off frequencies of the charge amplifier: *f*_L_ = $\frac{1}{2\pi R_{2}C_{2}}\;=\;8\;\mathrm{Hz}$ and *f*_H_ = $\frac{1}{2\pi R_{1}C_{1}}\;=\;200\;\mathrm{kHz}$, and *f*_C_ is the cut-off frequency of the low-pass filter: *f*_C_ = $\frac{1}{2\pi R_{3}C_{3}}\;=\;60.4\;\mathrm{Hz}.$

## 3. Results

### 3.1. Quality Assessment of ZnO NWs Grown on Paper

**Figure 3:**
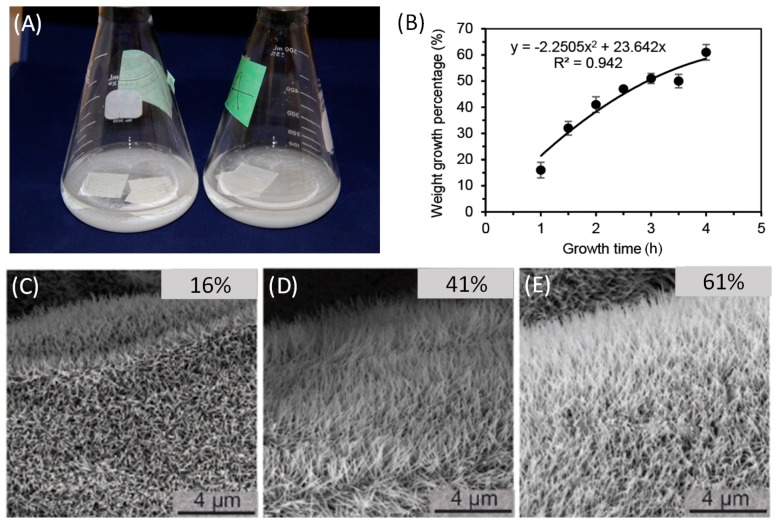
Hydrothermal Growth of ZnO NWs on cellulose paper. (**A**) Photograph of the hydrothermal growth setup in an 86 °C oven. (**B**) Calibration data of the weight growth percentage of the paper vs. growth time (*n* = 3). Note that the fitted second-order polynomial equation has no obvious physical meaning. (**C**–**E**) SEM photographs of the ZnO NWs after the growth periods of (**C**) 1 h, (**D**) 2 h and (**E**) 4 h. The average length of the ZnO NWs increases proportionally with the weight growth percentage.

The hydrothermal growth of ZnO NWs on Whatman grade 1 chromatography paper (3 mm *×* 3 mm) was performed in a stopper flask (Figure 3A). We investigated the effect of growth time on the weight growth percentage of ZnO NWs on paper. As illustrated in Figure 3B, in the 4-h period the growth speed of the ZnO NWs gradually slows down, and this growth profile can be explained by the gradual depletion of chemicals in the growth solution. In particular, the growth percentages after 1, 2 and 4 h are 16 *±* 3.0%, 41 *±* 3.0% and 61 *±* 3.0% (*n =* 3), as shown in Figure 3B. It is known that the length of ZnO NWs could be tuned by varying the time that the paper samples have been in the growth solution [38,39]. Through scanning electron microscopy (SEM) imaging of the ZnO-NW-coated paper (Figure 3C–E), we confirmed that the weight growth of the paper after growth proportionally correlates with the average length of the ZnO NWs. Moreover, it can be seen that the cellulose microfibers were uniformly covered by the ZnO NWs pointing radially outward.

### 3.2. Frequency Response Testing of the Paper-Based Accelerometer

**Figure 4:**
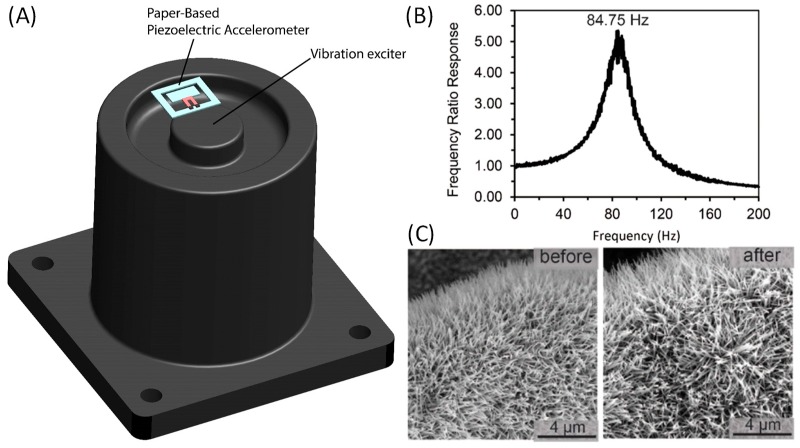
Frequency response characterization of the paper-based accelerometer. (**A**) Schematic of the experimental setup showing an accelerometer mounting on the electrodynamic exciter. (**B**) Frequency response curve of the accelerometer under random vibration testing. (**C**) SEM photographs of the ZnO NWs before and after the random vibration testing, showing no morphological degradation of the ZnO NWs.

The first step to calibrate the paper-based accelerometer was to measure the natural frequency of the device. A paper-based accelerometer was placed on top of an electrodynamic exciter (Type 4808, Brüel & Kjær, Nærum, Denmark) and a scanning laser Doppler vibrometer (PSV-400, Polytec, Waldbronn, Germany) was used to measure the proof mass vibration of the paper-based accelerometer and monitor its frequency response (Figure 4A). The exciter was set to undergo a random vibration testing so it excited all the frequencies at any given time. As shown in Figure 4B, the natural frequency of the paper-based accelerometer is 84.75 Hz. Note that, as the mass differences of the ZnO NWs under different hydrothermal growth periods are negligible comparing to the mass of paper device structures, the growth weight percentage of the ZnO NWs did not obviously affect the natural frequency of the accelerometer. Typically, the viable frequency range of accelerometer operation is within 20% of the measured natural frequency [40]. As a result, the working range for the paper-based accelerometer is within 16.95 Hz. This limited bandwidth is due to the low stiffness-to-mass ratio of the paper device structures. Moreover, we investigated if there was any morphological degradation of the ZnO NWs as the paper device underwent random vibration testing, by comparing the SEM photographs of the ZnO NWs before and after the test. As illustrated in Figure 4C, no obvious degradation (i.e., breakage and/or bending of the ZnO NWs) can be observed.

### 3.3. Comparison with Commercial MEMS Accelerometer

**Figure 5:**
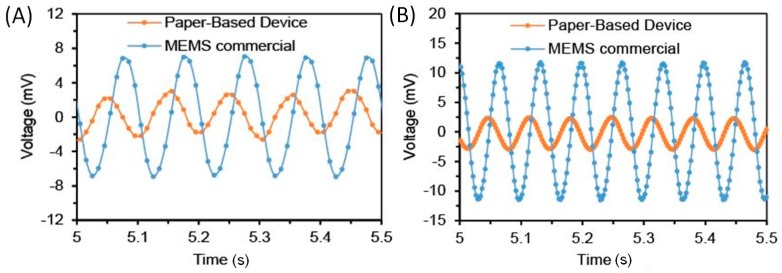
Comparison with commercial MEMS accelerometer. (**A**) Device outputs at sinusoidal excitation of 10 Hz. (**B**) Device outputs at sinusoidal excitation of 15 Hz.

We mounted a paper device (ZnO-NW weight growth percentage: 61%) and a commercial MEMS accelerometer (MMA 7341, Freescale Semiconductor, Austin, TX, USA) on the same electrodynamic exciter and let them undergo sinusoidal excitations at 10 Hz and 15 Hz. The MEMS accelerometer is integrated with its signal readout circuit and can directly output an analog voltage with a sensitivity of 117.5 mV/g. As shown in Figure 5A,B, at 10 Hz and 15 Hz excitations, both accelerometers generate output signals following sinusoidal waveforms. The magnitude of the voltage output from the MEMS accelerometer is higher than that from the paper-based accelerometer, demonstrating a higher sensitivity of the MEMS accelerometer at both frequencies. It was found that there is a phase lag of approximately 270° between the outputs of the paper-based accelerometer and the MEMS accelerometer. This phase difference was due to the different phase shifts caused by the signal readout circuits of the MEMS and paper-based accelerometers.

### 3.4. Sensitivity Measurement of the Paper-Based Piezoelectric Accelerometer

**Figure 6:**
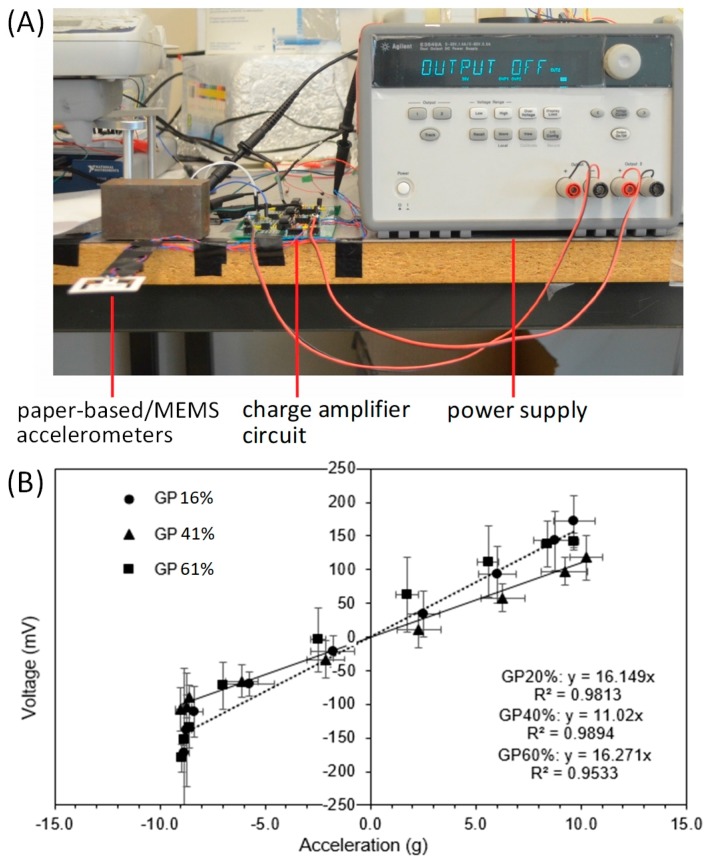
Sensitivity calibration of the paper-based accelerometer. (**A**) Experimental setup. (**B**) Calibration results of the device sensitivity with different ZnO-NW growth percentages (*n* = 3).

Finally, we measured the sensitivity of the paper-based accelerometers with three different weight growth percentages of ZnO NWs. For this test, a paper-based accelerometer and a MEMS accelerometer were attached to the top and bottom surfaces of the free end of a metal ruler, and the other end of the ruler was clamped to the surface of a table, as shown in Figure 6A. To exert accelerations to the two accelerometers, the free end of the ruler was deflected downward by 3.5 cm from its initial position and then released. The bending vibration of the released ruler applied oscillating accelerations to the accelerometers, and their output signals were measured by the DAQ board. The output of the MEMS accelerometer was used to calculate the applied acceleration and thus generate the calibration curves of the paper-based accelerometer. Figure 6B shows the calibration curves of the paper-based accelerometers with ZnO NWs at three different weight growth percentages (*n* = 3 devices for each percentage). We calculated the sensitivities of the paper-based accelerometers to be 16.1 mV/g, 11.02 mV/g and 16.3 mV/g for the weight growth percentages of 16%, 41% and 61%, respectively.

## 4. Discussion

In this work, we demonstrated the feasibility of acceleration measurement using ZnO-NW-coated cellulose paper as the major sensing element. The addition of acceleration sensing capability to common paper substrates is of primary importance to developing monolithic paper-based devices with a variety of sensing capabilities such as strain/touch sensing and energy harvesting. Our design has a few useful characteristics for applications in paper-based electronics. (i) The hydrothermal approach of growing ZnO NWs used in this work is compatible with many types of paper substrates, making our design applicable to a variety of paper-based electronic devices involving different types of paper. (ii) The hydrothermal synthesis of ZnO NWs could be spatially controlled by patterning the seeding layer on a substrate through screen/inkjet printing or other means; this allows the formation of ZnO-NW micro-patterns and thus leads to monolithic, highly-integrated and more versatile designs of paper-based sensors and electronics. (iii) The device fabrication process is low-cost and does not require the access to expensive, sophisticated equipment. Most importantly, the process is compatible with existing techniques for fabricating electronic devices on flexible substrates such as screen and inkjet printing. Roll-to-roll processes can also be adapted for paper cutting and electrode printing.

Prior to experimental testing of the paper-based accelerometers, we speculated that the longer the ZnO NWs (i.e., the higher its weight growth percentage) growth on paper, the more piezoelectric charges could be generated from the mechanical loading of the ZnO-NW-coated paper during acceleration measurement. However, the data suggested that there is no obvious correlation between the sensitivity of the paper-based accelerometer and the weight growth percentage of ZnO NWs on paper. A possible reason for this experimental observation is that the piezoelectric sensing mechanism of the ZnO-NW-coated paper involves multiple loading modes of the ZnO seeding layer and the individual ZnO NWs. There are two possible sources of the electronic charges produced from the ZnO-NW-coated paper: (i) deformation of the ZnO seeding layer on the paper microfibers when the microfibers are stretched and compressed; and (ii) surface rubbing and rubbing-induced deformation of adjacent ZnO NWs when their rooting paper microfibers are deformed. In addition to the unclear deformation modes of the ZnO NWs, the transport pathways of generated charges could also be significantly altered when adjacent ZnO NWs contact each other during device operation. All these intertwined factors explain the factor that the increase in ZnO-NW length does not necessarily lead to the enhancement of the device sensitivity. As the growth percentages of 16% and 61% lead to similar device sensitivities (16.1 mV/g vs. 16.3 mV/g; Figure 6B), fabricating the paper-based accelerometer at a growth percentage of 16% will be cost-effective. The sensitivity of the current paper-based accelerometer prototype would be sufficient for certain types of vibration-monitoring applications (e.g., for smart packaging) that require low-to-moderate sensitivity but ultralow device cost.

With the prototype devices, we have demonstrated the feasibility of our design for acceleration measurement. Further improvements on the performance and functionality of the paper-based accelerometer can be carried out. (i) In our current design, the ZnO NWs were synthesized on a separate piece of paper, which was then attached to the parallel paper beams as the strain gauge. Direct growth of the ZnO NWs on the parallel beams is also possible through patterning of the ZnO NPs on the parallel beams. This will avoid attaching another piece of ZnO-NW-coated paper onto the paper device structures and thus improve the fabrication yield and device reliability. (ii) For the same growth percentage, the sensitivity calibration data (Figure 6B) show relatively large variations of the device output voltage among different devices. The consistency of the device output at the sample growth percentage can be further improved by finely adjusting and optimizing hydrothermal growth parameters of the ZnO NWs and thus improving the ZnO-NW consistency from device to device. (iii) The charge amplifier circuit can be directly integrated into the paper substrate to form a completely monolithic device, through printing of electrical connections and attachment of surface mount components on the paper substrate.

The paper-based accelerometer is made from low-cost materials including paper and conductive inks, and can be mass-produced through cost-effective approaches such as die cutting of paper and screening printing of conductive inks. We demonstrated that the paper-based accelerometer is capable of monitor vibration reliably but has a lower sensitivity than a MEMS accelerometer. Despite its lower sensitivity, the paper-based accelerometer will find potential applications in areas such as smart packaging and paper-based electronics. For instance, packages of delicate equipment may require real-time vibration monitoring during transportation and paper-based electronic devices (e.g., smart greeting cards) could also employ paper-based accelerometers for user-device interaction.

## 5. Conclusions

This paper reports the development of a paper-based piezoelectric accelerometer integrating ZnO NWs. We first grew ZnO NWs on cellulose paper through a low-cost hydrothermal approach and then integrated the ZnO-NW-coated paper into a cantilever-based configuration of paper-based accelerometer. As the paper-based accelerometer underwent accelerations/vibrations, the piezoelectric ZnO-NW-coated paper on the cantilever beams experienced stretching or compressive deformations and thus produced electric charges that were converted into voltage outputs through a charge amplifier circuit. The paper-based accelerometer with a proof mass of 61 mg was calibrated to have a sensitivity up to 16.3 mV/g and a natural frequency of 84.75 Hz. This design has potential for use in smart packaging and integration of motion-sensing capability into paper-based flexible electronics.
